# Enhanced Water Vapor Transmission through Porous Membranes Based on Melt Blending of Polystyrene Sulfonate with Polyethylene Copolymers and Their CNT Nanocomposites

**DOI:** 10.3390/polym8050190

**Published:** 2016-05-12

**Authors:** Georgia Ch. Lainioti, Giannis Bounos, George A. Voyiatzis, Joannis K. Kallitsis

**Affiliations:** 1 Department of Chemistry, University of Patras, GR-265 04 Rio-Patras, Greece; glainioti@upatras.gr; 2 Foundation for Research and Technology Hellas-Institute of Chemical Engineering Sciences (FORTH-ICEHT), P.O. Box 1414, GR-265 04 Rio-Patras, Greece; iboun@iceht.forth.gr

**Keywords:** breathable membranes, melt blending, polystyrene sulfonate, carbon nanotubes, water vapor transmission rate

## Abstract

A novel concept for the use of an immiscible and non-meltable polymer, such as sodium polystyrene sulfonate (PSSNa), in order to prepare polyethylene non-woven breathable membranes is described. Membranes were fabricated by melt compounding of properly functionalized PE (P(E-*co*-AA)) and PSSNa (P(SSNa-*co*-GMA)) copolymers in the presence of water soluble polyethylene glycol (PEG). The inability of PSSNa derivatives to be melted was overcome by using PEG, which was easily meltable thus inducing PSSNa processability improvement. PEG was removed after membrane fabrication and therefore also acted as a porogen. Carbon nanotubes, functionalized with PSSNa moieties or alkyl groups, were also incorporated in the membranes with the aim of improving the porous connectivity and increasing the water vapor transmission rate. The morphology of the membranes was investigated through Scanning Electron Microscopy (SEM). Water vapor transmission rate (permeation) (WVTR) measurements for the porous membranes showed increased values in comparison with the neat PE ones. A further increase of WVTR was observed with the addition of CNTs to the polymer membranes.

## 1. Introduction

In recent years, the most common and abundant group of commercially available membranes is represented by polymeric membranes, as they have a wide field of applications, including chemical [1], food [2] and pharmaceutical industries [3], water treatment [4], *etc.* The necessity for membranes with different separation properties is growing the interest in this research field. Several polymeric matrices have been used for the synthesis of breathable membranes. Microporous polyurethane membranes or poly(acrylonitrile-*co*-maleic acid) membranes modified with poly(ethylene glycol) have been used as membrane materials [5,6]. Moreover, polyolefins are highly attractive materials due to their abundance and low cost. Microporous films and composites have been made by using polyolefin material and inorganic fillers [7]. Special engineering fibers and their fabrics can be combined with these microporous films to achieve a variety of properties for practical applications.

Hydrophilic polymers are interesting as membrane materials because of their reduced adsorption tendencies and high water (vapor) flux. Generally, a single membrane material hardly possesses all the desirable properties including film formation, good mechanical strength, thermal stability, chemical durability, *etc.*, at the same time. As a result, the achievement of a hydrophobic–hydrophilic balance emerges as a property of high importance, as it will improve the membrane performance and satisfy different requirements.

A well-known and simple technique to both accomplish this compromise and manufacture polymeric membranes is the production of polymer blends via melt-processing. The combination of two or more polymers via melt blending in order to obtain new polymers with improved properties is of utmost importance as it constitutes a practical and cost-effective method, although the challenge of their recycling grows. The compatibilization of immiscible polymers is of high scientific and industrial interest. The main strategies for that achievement are based (a) in the physical blending of the immiscible pairs with further addition of a pre-formed copolymer to compatibilize the blend (suitable block or graft copolymers that act as interfacial agents), and (b) in the reactive blending of relevant polymers bearing complementary reactive groups by the *in situ* generation of the required copolymers through polymer–polymer grafting reactions using functionalized polymers [8,9,10].

Despite the fact that many attempts have been made to maximize membrane performance by varying the molecular structure, polymers still exhibit a trade-off between permeability and selectivity [11]. However, due to the processing flexibility and low cost of polymers, polymeric membranes are still highly attractive for many industrial applications. Thus, efforts have been undertaken in order to use hybrid materials for the achievement of high permeability and selectivity in membrane applications [12,13].

Carbon nanotubes (CNTs) have attracted considerable attention from both academic and industrial sectors due to their outstanding properties making them promising materials in various fields. An approach that raised particular attention was the use of CNTs to control the water transport in membranes [14,15]. Many studies in the last few years suggest the use of nanotube networks and assemblies as a great tool for high efficiency water filtration [16,17,18]. However, for the development of polymer nanocomposites, the homogenous dispersion of CNTs in the polymer matrix to avoid aggregation problems is a prerequisite. For this reason, various functionalization procedures have been applied to CNTs to improve their compatibility with the polymer matrix [16] and, at the same time, to avoid any impact on their water permeability.

Experimental studies on the water transport properties of polymer/CNT or other inorganic filler in Mixed Matrix Membranes are scarce [19] and results are somewhat contradictory. Experimental investigation on CNT–PVA membranes indicates that the water transport rate increases with filler concentration as a consequence of the reduced crystallinity of the polymer matrix [20]. The opposite result was obtained in an experimental study on composites of multiwalled carbon nanotubes with segmented polyurethane (MWCNT–SPU) membranes where the decrease of water permeability with the increase of filler content was attributed to the increased stiffness of the polymer chains [21]. With the incorporation of another type of inorganic filler like montmorillonite (MMT) in Hydroxypropylmethylcellulose (HPMC), Polyvinyl alcohol (PVA) and methylcellulose/pectin nanocomposite films [22,23,24], similar behavior of decreasing the WVTR with the incorporation of filler was observed. This water vapor barrier elevation of polymer/clay composite films is mainly attributed to the tortuous paths of water vapor diffusion due to the impermeable clay layers distributed in the polymer matrix which increase the effective diffusion path length [25].

In the present study, polyethylene membranes were prepared through reactive blending of sodium polystyrene sulfonate–glycidyl methacrylate and ethylene–acrylic acid copolymers in the presence of polyethylene glycol (PEG). The key achievement here is that, with this methodology, we made the reactive blending between sodium polystyrene sulfonate copolymers and polyethylene or polypropylene matrices possible. After PEG removal, new porous membranes as novel polyethylene non-woven breathable films were obtained. Taking advantage from the unique transport properties of the CNTs, CNTs were also incorporated in such porous membranes with the aim of improving the porous connectivity and increasing the water vapor transmission rate. The main objective of this paper is to provide an alternative technique for making cost-effective microporous films using conventional polyolefin material. The significance of the method lies in the fact that, via a single step, the alteration of the PE porosity and the formation of interconnecting network among the microporous PE structure are simultaneously achieved. Most important, the expensive and limited standard procedure of the biaxial drawing, reported as a method to create an interconnecting network of microvoids into the mineral (CaCO_3_) filled PE films [26] produced by debonding at PE/mineral interfaces, has been avoided.

## 2. Materials and Methods

### 2.1. Materials

The monomers glycidyl methacrylate (GMA) and sodium styrene sulfonate (SSNa) and the initiator azobisisobutyronitrile (AIBN) were purchased from Sigma-Aldrich Co. (St. Louis, MO, USA) and used as received. The solvents *N*,*N*-dimethylformamide (DMF), acetone and deuterated chloroform (CDCl_3_) were purchased from Fischer Scientific (Pittsburgh, PA, USA) and used as received. Ultra-pure water was obtained by means of a SG apparatus water purification unit. Polymers poly(ethylene-*co*-acrylic acid) and polyethylene glycol and 1,2-dimethylimidazole (DMI), 98% were purchased from Sigma-Aldrich Co. Multi-walled carbon nanotubes (MWCNTs) of 97% purity as-produced (outer diameter of 15–35 nm) were provided by Nanothinx S.A (Rio-Patras, Greece). A commercial breathable membrane, 25 μm-thick (coded Celgard 2400), was kindly provided by Celgard LLC (Charlotte, NC, USA) for comparison in water vapor transmission tests. 

### 2.2. Synthesis and Characterization of Copolymer through Free Radical Polymerization

**Scheme 1:**
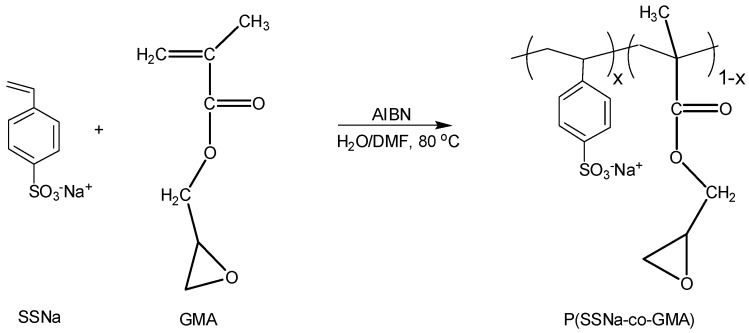
Reaction steps for the synthesis of the P(SSNa-*co*-GMA) copolymers.

The copolymers poly(sodium styrene sulfonate-*co*-glycidyl methacrylate) were synthesized through free radical polymerization in DMF/H_2_O, using AIBN as initiator. The synthetic procedure is illustrated in Scheme 1. The copolymer is denoted as P(SSNa*x*-*co*-GMA(1 − *x*)), where *x* is the mol fraction of SSNa units and (1 − *x*) is the mol fraction of GMA units, respectively, in the copolymer, as determined by ^1^H NMR characterization in D_2_O. Briefly, the desired quantity of the two monomers (total monomer concentration 1 M) was dissolved in the appropriate solvent, the solution was degassed, and the initiator AIBN (0.02 mol % over the total monomer concentration) was added. The reaction was left to proceed overnight under vigorous stirring in an Ar atmosphere in an oil bath set at 80 °C. After cooling down to room temperature, the copolymers were recovered by precipitation in acetone, filtered and dried in a vacuum oven at 60 °C for 24 h.

**Figure 1:**
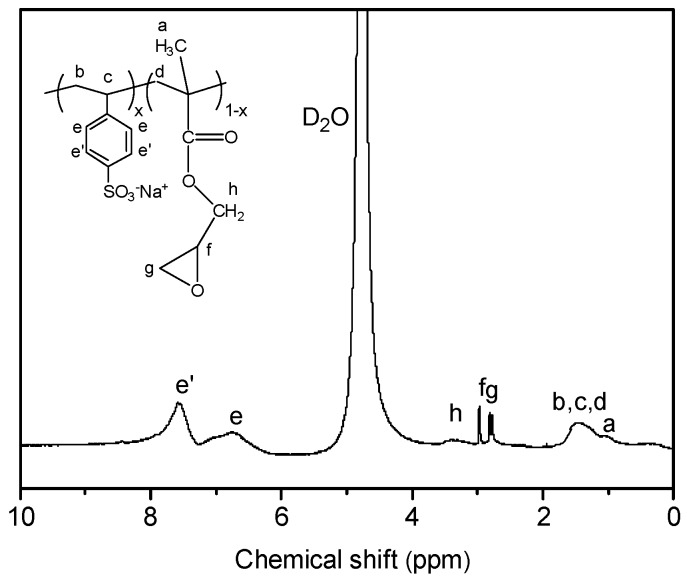
^1^H NMR spectrum of P(SSNa-*co*-GMA0.2).

A representative ^1^H NMR spectrum of the copolymer P(SSNa-*co*-GMA0.2) with a GMA content 20%, is illustrated in Figure 1, where the characteristic peaks of SSNa and GMA are observed. More specifically, the peaks at 6.0–8.0 ppm are assigned to the aromatic protons of SSNa. As far as GMA is concerned, signal originating from the methylene bonded to the ester oxygen was observed at 3.3 ppm (h type protons), the methine proton of the oxirane ring was observed at 2.9 ppm (f type protons), while two protons for the methylene of the ring were observed at 2.7–2.8 ppm (g type protons). The peak at 4.7 ppm is attributed to the deuterated water.

### 2.3. Surface Modification of Carbon Nanotubes

#### 2.3.1. Surface Polymerization of SSNa Monomer onto Carbon Nanotubes

MWCNTs were initially functionalized with hydroxyl groups via diazonium chemistry, as reported elsewhere [27,28], using 4-aminophenol and isopentyl nitrite (MWCNTs–OH). The hydroxyl-modified CNTs were then esterified with 2-chloropropionyl chloride for the attachment of initiator groups on the surface of nanotubes (MWCNTs-Init). Furthermore, surface-initiated ATRP was employed for the polymerization of hydrophilic sodium styrene sulfonate (SSNa) monomer onto modified CNTs (MWCNTs-*g*-PSSNa) [29,30].

#### 2.3.2. Functionalization of Carbon Nanotubes with Undecyl (C_11_-) Radicals

Based on a synthetic procedure mentioned elsewhere [31], in a 1000-mL round bottom flask, equipped with a magnetic stirrer, 2 g of MWCNTs were dispersed in 900 mL of toluene under argon. For the better dispersion of CNTs, the mixture was placed in an ultrasonic bath for 30 min. Additionally, 2 g of lauroyl peroxide was added. Temperature was raised to 80 °C. Next, 1 g portions of lauroyl peroxide were added, successively, every 2 h of synthesis. Total reaction time was 15 h, and overall weight of consumed lauroyl peroxide was 6 g. The obtained undecyl-functionalized MWCNTs (MWCNTs-C_11_) were vacuum filtered through Nylon membrane (200 nm). The obtained MWCNTs-C_11_ was washed several times with toluene and hexane.

### 2.4. Reactive Blending and Membrane Preparation

Before blending, polymers were dried at 60 °C, for 12 h in a vacuum oven. Blends of P(ethylene-*co*-AA), P(SSNa-*co*-GMA), PEG and DMI [32] were prepared in a mixer equipped with a cylindrical rotor at 180 °C, for about 20 min. A nitrogen blanket was used to minimize polymer degradation. First, a mixture of P(SSNa-*co*-GMA) with PEG and DMI was added to the mixer and left to melt for 2 min. P(ethylene-*co*-AA) was then added and the sample processed under a rotation speed of 100 rpm for 18 min. After blending, films were prepared by compression molding process, through an Electric Thermo hydraulic press, at 200 °C, between two Teflon sheets with a pressure of 15 MPa for approximately 3 min. After removal from the hot press, the samples were quenched by immersion in an ice-water bath. The resulting blend films were homogeneous with thickness that ranged from 70 to 200 µm. The epoxide/acid groups’ ratio was a crucial factor for the chemical reaction of the copolymers. For the incorporation of carbon nanotubes into the polymeric membranes, the above-mentioned procedure for the blend preparation was followed. The addition of carbon nanotubes was done at the last stage and the concentration was 1 wt % of the weight of P(ethylene-*co*-AA). For obtaining porous structure via PEG extraction, membranes were immersed in a water bath at 80 °C for 30 min.

### 2.5. Characterization Techniques

^1^H NMR spectra were obtained from a Bruker Avance DPX 400 spectrometer (Billerica, MA, USA), with DMSO-d6, containing TMS as internal standard, and D_2_O as solvent.

Thermogravimetric analysis (TGA) was carried out in alumina crucibles in a Labsys™ TG (Caluire, France) apparatus of Setaram under nitrogen and at a heating rate of 10 °C/min.

Scanning electron microscopy (SEM, Zeiss SUPRA 35VP instrument equipped with an EDS detector, Oberkochen, Germany) was performed to investigate the cross-section morphologies of the composite membranes. All membrane samples, frozen in liquid nitrogen, were broken and sputtered with gold to produce electric conductivity before SEM examination. The cross-section of the membranes at the broken parts was finally examined by SEM.

### 2.6. Water Vapor Transmission Rate

**Figure 2:**
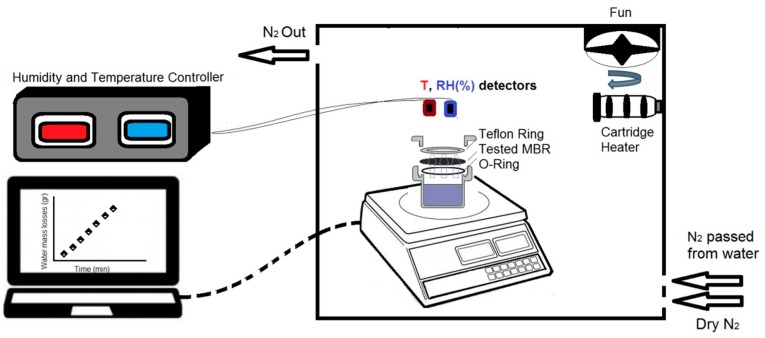
Homemade experimental setup used for water vapor transmission rate (WVTR) measurements.

The technique used to measure water vapor transmission rate (WVTR) was the wet cup method described by ASTM E96/E96M-10 [33]. According to this method, an acetal homemade dish filled with distilled water is covered by the tested membrane and placed in a chamber under controllable conditions of humidity and temperature [34]. The chamber consist of a homemade cartridge heater for temperature controlling, two inlets N_2_ for controlling the humidity (one for dry N_2_ and another one for N_2_ passed through water) and an axial fun for air circulation, as presented in Figure 2. During the experimental procedure the weight change of the complete test assembly is measured every 10 min by a computer-interfaced scale inside the chamber. The experimental conditions for all the examined membranes were 37 °C and 50% relative humidity (RH). Water vapor transmission rate (WVTR) is defined as the steady water vapor flow in unit of time through unit of area of a body, normal to specific parallel surfaces, under specific conditions of temperature and humidity at each surface. The WVTR was calculated from the steady-state region of the water losses time curves. The examined membrane area was *A* = 10 cm^2^. The slope of the water loss as a function of time normalized to the testing area *A* was taken as the water vapor transmission rate (WVTR):

(1) $$\text{WVTR}=\frac{\text{Mass}\;H_{2}O\;\text{lost}}{\text{time}\times \text{area}}$$

with units of g·m^−2^·min^−1^. Since the thickness of the films varied, the *WVTR* was normalized to film thickness *l* in order to obtain the specific water vapor transmission rate (*l* × WVTR) with units of μm·g·m^−2^·min^−1^.

## 3. Results and Discussion

### 3.1. Surface Modification of Carbon Nanotubes

**Figure 3:**
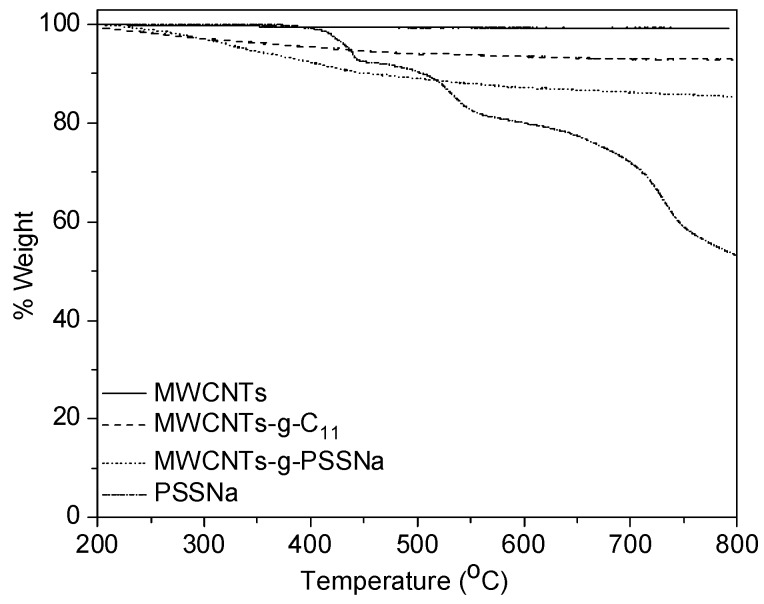
TGA analysis of pristine MWCNTs and MWCNTs functionalized with the undecyl groups (MWCNTs-C_11_) and the sodium styrene sulfonate monomer (MWCNTs-*g*-PSSNa).

PSSNa-*g*-MWCNTs hybrids were prepared, as previously described [28], using atom transfer radical polymerization of SSNa on CNTs-initiator [27], derived from MWCNTs functionalized with hydroxyl groups. Grafting polymerization of sodium styrene sulfonate monomer took place from the surface of initiator-functionalized carbon nanotubes. Functionalizations were confirmed with thermogravimetric analysis, as shown in Figure 3.

The TGA results indicated that the mass losses of MWCNTs–OH and MWCNTs-Init were approximately 6% and 8% (data not shown), compared to pristine MWCNTs, which is an indicator of successful functionalization. Moreover, an additional weight loss of 4% for MWCNTs-*g*-PSSNa, compared to MWCNTs-Init, reveals the polymerization of sodium styrene sulfonate (SSNa) monomer onto the surface of CNTs-Init through ATRP. In another approach, MWCNTs were functionalized with undecyl (C_11_-) radicals generated by thermal decomposition of lauroyl peroxide. It is well known that one of the best methods for achieving homogeneous dispersion of CNTs in a polymer matrix is the functionalization of the MWCNTs [35,36]. The functionalization of CNTs with moieties that are structurally close to the polymer matrix ensures the compatibility of the dispersed nanomaterials with the matrix [37]. The successful functionalization was verified from TGA analysis, as shown in Figure 3. The TGA results demonstrate a sufficient weight loss of 6.5% for MWCNTs-C_11_ compared to pristine MWCNTs, which correspond to the thermal disruption of the alkyl attachments.

### 3.2. Chemical Structure of Membranes

Development of breathable membranes by melt blending procedure is a demanding topic since it requires the presence of a continuous matrix for mechanical integrity and controlled porosity in the presence of hydrophilic functionality for water vapor interactions and transport. Moreover, the combination of a polar polymer, like PSSNa, with polyolefins results in low quality films due to their inherent immiscibility and the PSSNa inability to melt. In an attempt to overcome these limitations, we used properly functionalized PE (e.g., P(E-*co*-AA)) and PSSNa (P(SSNa-*co*-GMA)) for the fixation of the hydrophilic PSSNa groups onto the polyolefin backbone through reactive blending.

In this study, melt blending of ethylene-acrylic acid and sodium styrene sulfonate-glycidyl methacrylate copolymers was done with the use of 1,2-dimethylimidazole (DMI) as a catalyst. Imidazoles have been reported as efficient catalysts for the epoxy/acid reaction, especially in commercial epoxy resin systems [38,39]. Our intent was obviously to achieve compatibilization of the two copolymers and obtain homogenous films. The chemical reaction between acrylic acid groups (–COOH) of P(E-*co*-AA) and glycidyl methacrylate groups of P(SSNa-*co*-GMA) resulted in the generation of an in-situ graft copolymer during the melt blending. The inability of PSSNa derivatives to be melted was overcome by using PEG that was easily meltable inducing thus the PSSNa processability improvement. PEG was added in order to reduce the melting temperature of the polymer, and also to act as a porogen, since it was removed during the water treatment of the membranes. Compositions of the blends and the water loss after PEG removal are collected in Table 1. Film quality for all examined compositions was excellent.

**Figure 4:**
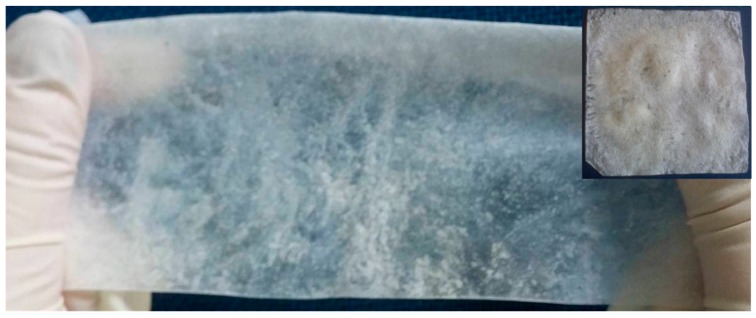
Membrane with P(ethylene-*co*-AΑc28)/P(SSNa-*co*-GMA0.2)/PEG composition 60/10/30 after the total removal of the water soluble polymer (PEG).

It can be observed there that PEG has been almost entirely removed from the majority of the membranes. However, in order to find the optimal conditions for the removal of PEG, immersion time of the membrane *versus* temperature of the water bath were examined. It is worth mentioning that PEG can be extracted at 24 h in the case of 40 °C, whereas at 80 °C the time can be reduced to 10–20 min. This provides the possibility to create controlled porosity in the membranes by changing the temperature value or/and the time of immersion. Figure 4 depicts the membrane with composition 60/10/30 after the total removal of the porogen.

For the preparation of the above-mentioned membranes, several parameters were examined, such as the mixing duration of the materials, the order of addition of the polymers, the ratio of the molar equivalents of active groups (acid/epoxide ratio), the percentage of GMA (10% or 20%) in the P(SSNa-*co*-GMA) copolymer and the presence or absence of a catalyst. An optimization of the acid/epoxide ratio was held in order to find the best film quality. Moreover, a large quantity of DMI is necessary to activate the grafting reaction (moles of DMI > 20 mol of –COOH) [32].

Then, experiments were conducted in order to integrate modified carbon nanotubes in the PE-*g*-PSSNa membranes. For this purpose, mixtures of the polymers P(ethylene-*co*-AA), P(SSNa-*co*-GMA), PEG and modified carbon nanotubes in different compositions were prepared, which are presented in Table 2. The modification of the MWCNTs was performed with the water-soluble polymer PSSNa (MWCNTs-*g*-PSSNa), as well as the hydrophobic groups C_11_ (MWCNTs-*g*-C_11_). The films, as shown in Table 2, had very good homogeneity. Films were then immersed in distilled water for PEG removal and pore formation. Film quality for all examined compositions was excellent.

### 3.3. Morphology of the Blends

**Figure 5:**
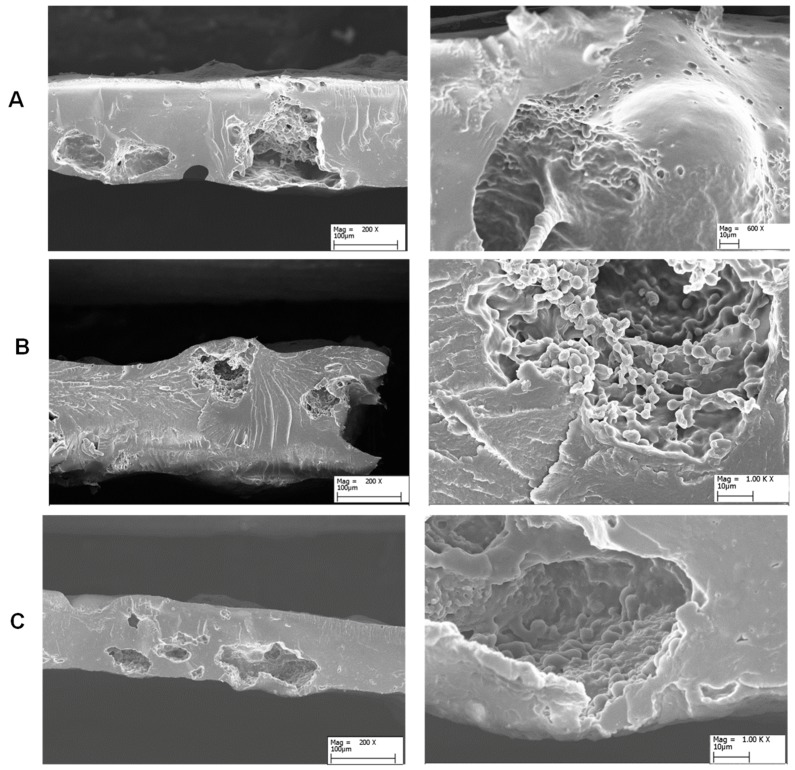

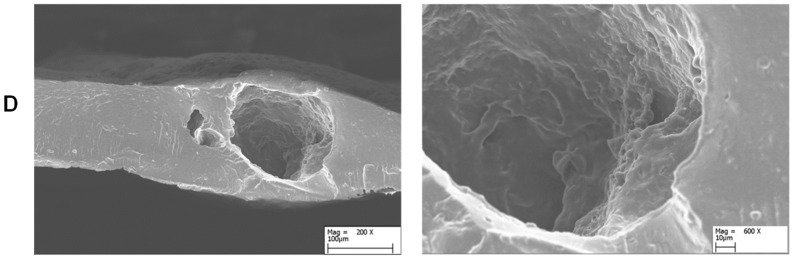
SEM images in two different magnifications of the cross-section morphology of PE-*g*-PSSNa membranes with various blend compositions: (**A**) M1; (**B**) M3; (**C**) M4; (**D**) M5.

The morphological characterization of the membranes, after the removal of the water soluble polymer and the formation of the porous structure, was done with scanning electron microscopy (SEM). Figure 5 shows SEM photographs of the cross section of membranes with various blend compositions produced using DMI as a catalyst. Cross-sectional SEM images of the composite membranes exhibit a porous structure at the surface skin layers of the membranes, in the range of μm. Moreover, it is worth mentioning that some macro voids have been appeared, revealing the inner structure of the membrane. In some cases, a uniform matrix has been developed, which is a strong evidence of the reaction of the polymers bearing reactive groups. The SEM micrographs evidently showed that after the melt blending of polymers and the removal of the water soluble PEG, a porous structure was created on the membranes with some limited interconnected pathways.

### 3.4. TGA Characterization

**Figure 6:**
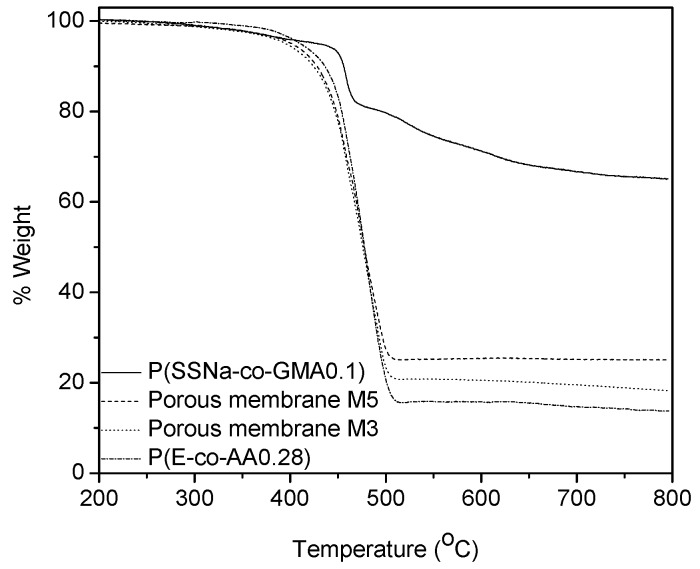
TGA analysis of the PE-*g*-PSSNa porous membranes and the copolymers P(ethylene-*co*-AΑ0.28) and P(SSNa-*co*-GMA0.1).

The films after the removal of PEG were characterized with thermogravimetric analysis (TGA), as shown in Figure 6. TGA analysis was conducted under a nitrogen atmosphere. The heating rate was 20 °C/min and the final temperature 800 °C. In Figure 6, a high initial decomposition temperature was observed at 460 °C, which appeared in all the porous synthesized membranes. Moreover, an intermediate weight loss was observed for the porous membranes, compared with the TGA graphs of the copolymers. The residue of M3 and M5 at 500 °C was 21% and 25%, respectively, whereas the residue of the copolymers P(SSNa-*co*-GMA0.1) and P(E-*co*-AA0.28) was, in that order, 78% and 15%, respectively. Bearing in mind these values, as well as the initial blend composition for each membrane (the percentage of P(E-*co*-AA0.28)/P(SSNa-*co*-GMA0.1) was 60/10 for M3 and 50/15 for M5), the theoretical weight residue for the porous membranes could be estimated. In this context, the residue determined by the copolymers was 25% for M3 and 30% for M5; these values are in line when compared to the experimental ones.

### 3.5. Water Vapor Transmission

**Figure 7:**
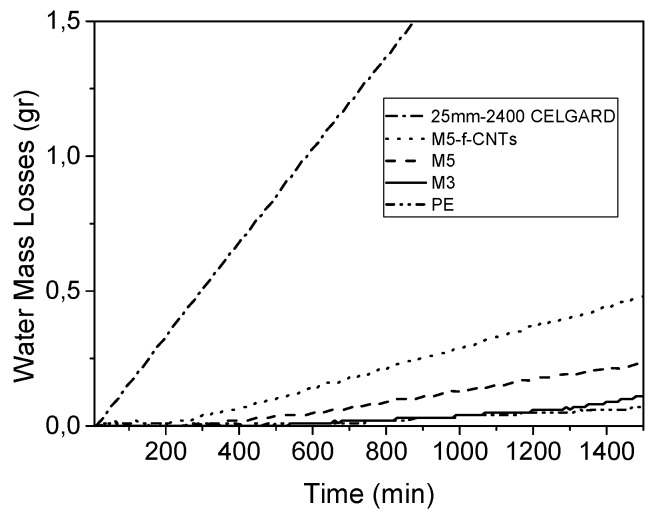
Typical curves of water mass losses as a function of the tested time.

Water vapor transmission measurements were performed on porous PE-*g*-PSSNa membranes obtained after PEG removal. Membranes with various compositions, with and without the incorporation of functionalized CNTs were tested. The water vapor transmission of these membranes was compared with the corresponding of pure polyethylene membrane and a commercially available porous membrane, the 25 μm-thick Celgard 2400 [40]. Figure 7 depicts representative curves showing the time dependence of water mass losses of pure PE membrane, and porous membranes M3, M5 and M5-*f*-CNTs(C_11_). For all the composite membranes, the rate of water loss was linear with time after an initial period of about 2 h. This initial period was attributed to temperature equilibration in the sample dish. Furthermore, all the composite membranes exhibit negligible swelling of ~7% during the test procedure.

Specific water vapor transmission rates (Sp.WVTR) were calculated from the linear region of the curve. The above-mentioned values, along with the composition and membrane thickness, are presented in Table 3. The extracted Sp.WVTR value for pure PE was found to be 1.94 μm·g·m^−2^·min^−1^. This value is in good accordance with the literature value for PE [41]. For the porous membranes M3 and M5, the Sp.WVTR values were 11.18 and 18.05 μm·g·m^−2^·min^−1^, respectively. It is worth mentioning that in general, the transmission of the water vapor is strongly related to the porous structure as well as to the hydrophilic groups contained in the membranes. Thus, in the present study, the transmission may be affected not only by the PEG content, the removal of which creates the porous structure, but also by the percentage of the hydrophilic copolymer P(SSNa-*co*-GMA) in the membrane composition. As a result, the slight improvement in the Sp.WVTR value appeared at the M5 membrane is owed to the above mentioned parameters.

Moreover, the incorporation of undecyl-functionalized MWCNTs (MWCNTs-C_11_) in PE-*g*-PSSNa and the subsequent PEG removal, led to the formation of the M5-*f*-CNTs(C_11_) type membrane with a substantial increase in the Sp.WVTR value to 53.13·μm·g·m^−2^·min^−1^, as shown in Table 3 for the M5-*f*-CNTs(C_11_) membrane; this value is increased by a factor of ~2.9 compared to the value of the M5 (CNTs free) membrane. From a theoretical point of view, considering the non-porous domain of M5-*f*-CNTs as a Mixed Matrix Membrane (MMM), these results can be explained by describing the MMM as a two-phase system consisting of a polymer matrix with a second pseudo-dispersed phase exhibiting enhanced water vapor permeability [42]. The second phase is most likely composed of MWCNTs with surrounding matrix interphase region. Interphase pores also act as fast diffusion channels, where the transport of penetrant molecules follow Knudsen diffusion, described by the following equation:

(2) $$D_{k}=\left(d_{p}/3\right){\left(8RT/m\right)}^{1/2}\left(1-\sigma_{\text{mol}}/2d_{p}\right)$$

where, *d*_p_ is the pore diameter, while *m* and *σ*_mol_ are the mass and size of the penetrant molecule, respectively [43]. The enhanced water permeability of this second phase can be explained considering the specific properties of the water–CNT system as reported in the literature in a theoretical and experimental context. Enhanced “apparent” water solubility is a consequence of water vapor condensation on the nanotube surface, as suggested by molecular dynamic studies [44,45] and/or of a “vapor-liquid” phase transition by capillary effects at the nanotube interior, as experimentally observed by TEM analysis [46]. When formation of liquid water takes place, CNTs act as a fast pathway for these penetrant molecules: the atomic-scale smoothness of CNT walls facilitates molecular ordering phenomena inside the tube, which weaken the carbon–water interactions and establish a friction-less transport mechanism [47]. The exact underlying mechanism for water transport and the further quantitative analysis, however, is difficult to be explained in this stage.

As already mentioned, the covalent functionalization of CNTs stands as a tool for obtaining both improved dispersion of CNTs and enhanced compatibility between polymers and nanotubes. This is due to the intermolecular interactions between polymer chains and functional groups on the surface of CNTs. The attachment to CNTs of alkyl chains that resemble the chemical structure of polyolefins is expected to reveal efficient interaction between nanocomposites and the polymer matrix.

As a result, the incorporation of functionalized CNTs in the membranes leads to the improvement of the continuity of porous pathways. The low interfacial force between hydrophilic water molecules and smooth hydrophobic CNT’s inner walls contributes to fast water vapor transport directly through the inner tubes of CNTs [48,49,50]. It seems that water molecules instinctively flow into the internal CNTs by forming a one-dimensional chain due to its higher thermodynamical stability within CNTs [14,51].

Comparing the Sp.WVTR of M5-*f*-CNTs(C_11_) and 25 μm-thick Celgard 2400 membrane, we realized that the values are quite similar. It is worth noting that 2400 Celgard membrane is formed by film extrusion, annealing and stretching of polypropylene and shows a three dimensional microporous structure [52]. Here, we also show that by a melt blending-manufacturing-process, the increased water vapor transmission performance of M5-*f*-MWCNT-C_11_ membrane points out the potential of such composite membranes for advanced water vapor transport applications.

## 4. Conclusions

In the present work, the incorporation of an immiscible and non-meltable polymer, like PSSNa, into a polyolefin matrix was achieved, through melt blending mediated by PEG. For this purpose, properly functionalized PE (e.g., P(E-*co*-AA)) and polystyrene sulfonate–glycidyl methacrylate copolymers were used. The integration of PSSNa to the PE matrix was done with the aid of the hydrophilic polymer PEG, which was subsequently removed to gain porosity in the membranes. In a further step, carbon nanotubes that have been successfully functionalized with PSSNa moieties or alkyl chain were incorporated to the membranes. As a result, a novel series of hydrophilic polymer membranes and hybrid polymer membranes were developed and their specific water vapor transmission rate (Sp.WVTR) was determined. The Sp.WVTR measurements for the porous membranes showed increased values in comparison with neat PE membranes. The incorporation of carbon nanotubes, functionalized with undecyl groups, enhanced the Sp.WVTR values revealing expectations toward breathable membrane applications.

## Figures and Tables

**Table 1:** Composition of blends and water loss of P(ethylene-*co*-AΑ0.28)/P(SSNa-*co*-GMA0.1 or 0.2)/PEG.

Membrane code	Polymers	Composition (wt %)	Loss (%)
M1	P(ethylene-*co*-AΑ0.28)/P(SSNa-*co*-GMA0.2)/PEG	60/10/30	28
M2	P(ethylene-*co*-AΑ0.28)/P(SSNa-*co*-GMA0.2)/PEG	50/10/40	20
M3	P(ethylene-*co*-AΑ0.28)/P(SSNa-*co*-GMA0.1)/PEG	60/10/30	28
M4	P(ethylene-*co*-AΑ0.28)/P(SSNa-*co*-GMA0.1)/PEG	50/10/40	40
M5	P(ethylene-*co*-AΑ0.28)/P(SSNa-*co*-GMA0.1)/PEG	50/15/35	35

**Table 2:** Composition of blends and water loss of P(ethylene-*co*-AΑc28)/P(SSNa-*co*-GMA0.1)/PEG and functionalized MWCNTs.

Membrane code	Functionalized MWCNTs	Composition (wt %)	Loss (%)
M3-*f*-CNTs(PSSNa)	MWCNTs-*g*-PSSNa	60/10/30	28
M4-*f*-CNTs(PSSNa)	MWCNTs-*g*-PSSNa	50/10/40	25
M5-*f*-CNTs(PSSNa)	MWCNTs-*g*-PSSNa	50/15/35	30
M3-*f*-CNTs(C_11_)	MWCNTs-*g*-C_11_	60/10/30	28
M4-*f*-CNTs(C_11_)	MWCNTs-*g*-C_11_	50/10/40	38
M5-*f*-CNTs(C_11_)	MWCNTs-*g*-C_11_	50/15/35	33

**Table 3:** Specific water vapor transmission rate (Sp.WVTR) values of the examined membranes.

Sample description	Composition (wt %)	Thickness (μm)	Sp.WVTR (μm·g·m^−2^·min^−1^)
PE	Pure	40	1.94
Celgard 2400	–	25	42.88
M3	60/10/30	70	11.18
M5 *	50/15/35	260	18.05
M5-*f*-CNTs(C_11_) *	49/35/15/1	150	53.13

* For data reproducibility three identical M5 membranes of different batches and two identical M5-*f*-CNTs membranes of different batches were tested.
